# Precision Medicine and Public Health: New Challenges for Effective and Sustainable Health

**DOI:** 10.3390/jpm11020135

**Published:** 2021-02-16

**Authors:** Deborah Traversi, Alessandra Pulliero, Alberto Izzotti, Elena Franchitti, Licia Iacoviello, Francesco Gianfagna, Alessandro Gialluisi, Benedetta Izzi, Antonella Agodi, Martina Barchitta, Giovanna Elisa Calabrò, Ilda Hoxhaj, Michele Sassano, Luca Gino Sbrogiò, Annamaria Del Sole, Francesco Marchiori, Erica Pitini, Giuseppe Migliara, Carolina Marzuillo, Corrado De Vito, Manuela Tamburro, Michela Lucia Sammarco, Giancarlo Ripabelli, Paolo Villari, Stefania Boccia

**Affiliations:** 1 Department of Public Health and Pediatrics, University of Torino, Piazza Polonia 94, 10126 Torino, Italy; elena.franchitti@edu.unito.it; 2 Department of Health Sciences School of Medicine, University of Genoa, 16132 Genova, Italy; alessandra.pulliero@unige.it; 3 Department of Experimental Medicine, University of Genoa, 16132 Genova, Italy; Alberto.Izzotti@unige.it; 4 IRCCS Ospedale Policlinico San Martino, 161632 Genova, Italy; 5 Research Center in Epidemiology and Preventive Medicine (EPIMED), Department of Medicine and Surgery, University of Insubria, 21100 Varese, Italy; licia.iacoviello@uninsubria.it (L.I.); francesco.gianfagna@uninsubria.it (F.G.); 6 Department of Epidemiology and Prevention, IRCCS NEUROMED, 86077 Pozzilli, Italy; alessandro.gialluisi@moli-sani.org (A.G.); benedetta.izzi@moli-sani.org (B.I.); 7 Mediterranea Cardiocentro, 80122 Napoli, Italy; 8 Department of Medical and Surgical Sciences and Advanced Technologies “GF Ingrassia”, University of Catania, 95123 Catania, Italy; agodia@unict.it (A.A.); martina.barchitta@unict.it (M.B.); 9 Section of Hygiene, University Department of Life Sciences and Public Health, Università Cattolica del Sacro Cuore, 00168 Roma, Italy; alisacalabro@icloud.com (G.E.C.); ilda.hoxhaj1@unicatt.it (I.H.); michele.sassano@unicatt.it (M.S.); Stefania.Boccia@unicatt.it (S.B.); 10 Dipartimento di Prevenzione, Az. ULSS3 Serenissima, 30174 Venezia, Italy; LucaGino.Sbrogio@aulss3.veneto.it; 11 Dipartimento di Prevenzione, SIAN, Az. ULSS5, 45100 Rovigo, Italy; annamaria.delsole@aulss5.veneto.it; 12 Azienda Ospedaliera Universitaria Integrata di Verona, 37126 Verona, Italy; francesco.marchiori@aovr.veneto.it; 13 Department of Public Health and Infectious Diseases, Sapienza University of Rome, 00185 Roma, Italy; erica.pitini@uniroma1.it (E.P.); giuseppe.migliara@uniroma1.it (G.M.); carolina.marzuillo@uniroma1.it (C.M.); corrado.devito@uniroma1.it (C.D.V.); paolo.villari@uniroma1.it (P.V.); 14 Department of Medicine and Health Sciences “Vincenzo Tiberio”, University of Molise, 86100 Campobasso, Italy; manuela.tamburro@gmail.com (M.T.); sammarco@unimol.it (M.L.S.); ripab@unimol.it (G.R.); 15 Department of Woman and Child Health and Public Health-Public Health Area, Fondazione Policlinico Universitario A. Gemelli IRCCS, 00168 Roma, Italy

**Keywords:** public health genomics, genetic polymorphisms, epigenetic modulations, miRNA, genetic and microbiome markers, health technology assessment, early disease prevention

## Abstract

The development of high-throughput omics technologies represents an unmissable opportunity for evidence-based prevention of adverse effects on human health. However, the applicability and access to multi-omics tests are limited. In Italy, this is due to the rapid increase of knowledge and the high levels of skill and economic investment initially necessary. The fields of human genetics and public health have highlighted the relevance of an implementation strategy at a national level in Italy, including integration in sanitary regulations and governance instruments. In this review, the emerging field of public health genomics is discussed, including the polygenic scores approach, epigenetic modulation, nutrigenomics, and microbiomes implications. Moreover, the Italian state of implementation is presented. The omics sciences have important implications for the prevention of both communicable and noncommunicable diseases, especially because they can be used to assess the health status during the whole course of life. An effective population health gain is possible if omics tools are implemented for each person after a preliminary assessment of effectiveness in the medium to long term.

## 1. Introduction

In recent years, statistical genetics and bioinformatics have made progress in the prediction of risk for different complex disorders, i.e., those diseases characterized by several genetic and environmental influences [1]. In particular, genomic markers have shown relatively high predictive power, and promising results are coming from other omics such as epigenomics, transcriptomics, proteomics, and metabolomics. The potentials for interactions among all these biomarkers and environmental data make us hopeful of finding cost-effective and clinically useful novel risk assessment tools for noncommunicable diseases [2].

Genetic susceptibility and environmental exposure alone are not sufficient to explain the pathogenesis of noncommunicable diseases, but should be integrated into a more complex scenario that can manifest pathological phenotypes. Epigenetics is a crucial component of this scenario, as its variations are linked to specific exposures that might be able to highlight the effects of the environment on the genome [3].

Scientific evidence has established that a healthy diet enhances life expectancy and helps prevent diseases. Although in the last few decades there has been huge growth in knowledge of the relationship between dietary components and diseases, the biological mechanisms underlying these effects are not yet well understood [4]. Nutritional epidemiology has contributed significantly to modern public health research and has promoted wellbeing and extended life expectancy. Specifically, researchers have investigated how the main social and behavioral determinants associated with the adherence to a healthy diet, such as the Mediterranean diet, could be useful in understanding and counteracting the global shift toward unhealthy patterns, to promote health and a better quality of life, especially in women of reproductive age [5,6].

Another emerging health determinant is microbiota status. The human microbiota consists of a complex ecosystem of microorganisms that live in the human body. Most of the microbial population is in the gut [7]. The ratio between the number of bacterial and human host genes is about 200:1, and such evidence can have profound effects on host phenotypes, playing critical roles in the host physiology [8]. The gut microbiota influences the evolution of adaptive phenotypic plasticity in mammalian species through the amplification of signals from the external environment, mainly during postnatal development. Therefore, such omics tools have become a crucial element in the risk stratification of the population for high-incidence diseases.

The increasing development of genetic tests has made the evaluation of their risks and benefits crucial to their appropriate translation into clinical practice, especially in the case of the COVID-19 pandemic [9]. The importance of a solid evaluation strategy for this kind of technology has been recognized across Europe and worldwide [10,11,12].

In this review, an overview of the public health implications is given, focusing on the Italian context. The building of health professionals’ and citizens’ omics sciences literacy is discussed, as well as the different regional departments’ perspectives.

## 2. Development and Perspectives for Italian Public Health Genomic and Epigenomic Tools 

### 2.1. Promising Perspectives from Clinical Genetics: The Use of Polygenic Scores and Epigenetic Markers

#### 2.1.1. Genetics

In the last few decades, genome-wide association scans (GWAS) have been used to identify the genetic basis of chronic diseases, which revealed the influence of several common genetic variants on their risk. These conditions include but are not limited to cardiovascular disease (CVD) [13,14], cancer [15], and metabolic [16], neurodegenerative [17], and neuropsychiatric disorders [18]. However, despite the high heritability observed for these chronic diseases, the genetic variants identified explained a small proportion of the disease variability. While waiting for novel and larger-scale studies allowing for the analysis of rare variants and numerous gene‒gene interactions with machine learning algorithms, many studies analyzed the potential clinical utility of the available variants in disease risk assessment.

Since these variants usually show a small effect size, they are generally condensed into polygenic scores (PGS), also known as polygenic risk scores, when dealing with diseases [19]. These are defined as quantitative factors aggregating the genetic influences of many common genetic variants on a single trait or disease. PGS has usually been computed as the sum of risk alleles that an individual has, weighted by the risk allele effect sizes, as estimated by an independent training GWAS on the phenotype (i.e., Beta or log (Odds Ratio), depending on the phenotype tested). Indeed, PGS represents an estimate of an individual’s genetic liability to a trait/disease, calculated according to their genotype profile, using data from independent GWAS as a training model [20]. Thanks to this construction, some PGS have been shown to explain a relatively high amount of the variance in continuous traits [21], like height, with a PGS explaining ~24% of the population phenotypic variance [22]. On the other hand, the variance explained by genetic predisposition to specific disorders is not as high, ranging from <2% for stroke [23] to 7% for schizophrenia [24], although this has revealed interesting genetic overlaps among disorders [25].

As a consequence, the integrated use of PGS with other risk algorithms using environmental factors has been proposed to predict the risk of chronic conditions in common clinical practice [26]. Among the chronic diseases, CVD appears to be the condition with the largest set of data and with potentials for the implementation of a cost-effective PGS [27]. Currently, the clinical guidelines for CVD primary prevention do not recommend the use of genetic data for risk assessment, since all PGS found to have high predictive power failed to modify treatments in a cost-effective manner or to motivate subjects to change their lifestyles [28,29]. Although a significant net risk reclassification improvement of subjects who will develop a particular condition was observed in most large and recent studies [30,31], cost‒benefit analyses should be performed to confirm their clinical utility for risk assessment. The number of subjects needed should be considered, along with the cost of biomarker measurements and the treatment of false-positive subjects [32]. The high laboratory costs and the relatively low added predictive power are currently the main barriers.

#### 2.1.2. Epigenetics

Epigenetic biomarkers represent a means by which lifestyle and environment can be taken into account in the study of health and disease [33,34,35]. DNA methylation, among all epigenetic markers, is very stable and, in principle, can be studied with no special experimental requirements [36] in both fluid and tissue specimens already in use in clinical practice [37,38,39,40,41]. DNA methylation markers are robust, sensitive, and measurable across individuals as well as in population studies. Unfortunately, the progress from preclinical observations to clinical translation of epigenetic biomarkers for noncommunicable diseases is still far from complete [42]. However, there is an interest in finding epigenetic biomarkers, especially in those clinical conditions where a traditional diagnosis fails in the identification of specific cases or where early prediction of the disease leads to a better prognosis or drug response.

The field of oncology is so far the most developed in terms of epigenetic biomarkers. A recognized signature of most cancer types is genome-wide hypomethylation, a hallmark of genome instability, alongside gene-specific hypermethylation (i.e., the silencing of tumor suppressor genes) [43]. The latter clearly defines cancer’s specificity and helps with differential diagnosis [44,45,46,47,48,49]. Among the few FDA-approved epigenetic tests, the screening of early colorectal cancer (CRC) is most frequently used in clinical practice, though it has lower specificity and higher costs than the traditional CRC screening tests [50,51].

Epigenetics is known to play a role in both neurodegenerative and neuropsychiatric diseases, as DNA methylation changes have been described in Alzheimer’s disease [52], Parkinson’s disease [53], amyotrophic lateral sclerosis [54], schizophrenia [55], major depressive disorder [56], and post-traumatic stress disorder [57]. Multiple studies have also shown the importance of epigenetics in the pathophysiology of type 2 diabetes (T2D) by studying the DNA methylation profiles in several relevant T2D target tissues [58,59,60], as well as using them for monitoring disease reversion after lifestyle intervention [61,62]. DNA methylation biomarkers in CVD are also far from being implemented into the clinical routine. A large number of epigenome-wide association studies have been performed, but with little reproducibility across cohorts. Despite this evidence, it is worth mentioning a few genes that have been found to have abnormal methylation patterns in association with both CVD and CVD risk factors, such as *GNAS* [63,64,65,66,67], *ZBTB12* [68,69], *BRCA1*, and *CRISP2* [70]. The discovery of these genes could pave the way for future studies along the same lines, possibly serving as good prediction biomarkers of CVD.

One important limitation on the use of epigenetic biomarkers in clinical practice is the tissue-specificity of the methylation patterns, so ideally the target tissue of the disease should be used. Despite this limitation, more and more studies are testing circulating blood as surrogate tissue for epigenetic analysis. Blood is a good source of DNA, obtained from noninvasive liquid biopsy, and is already used for epigenetic biomarker design in clinical practice for many human diseases [33,71]. DNA can be extracted from whole blood, peripheral blood mononuclear cells (PBMCs), single white blood cell (WBC) types, plasma (i.e., exosomes and free circulating-DNA and RNA), or platelets (mitochondrial). Recently, extracellular microRNAs (miRNAs) have been detected in biological fluids and studied as possible cancer markers that can be detected by noninvasive procedures [72].

Very recently, cell-free DNA (cfDNA) epigenetic research has been gaining more attention as a possible means to identify and predict tissue damage or the origin of certain pathological conditions [73]. CancerLOCATOR is a cfDNA-based method that allows for detection and predicts the tissue of origin using CpG methylation profiles [74]. Another study demonstrated that cfDNA methylation profiles were able to identify cardiomyocyte death [75], a possible useful marker of cardiac injury after myocardial infarction. Taken together, these findings suggest a possible use of these markers in clinical practice to predict and/or diagnose a clinical condition, thus better informing further disease prognosis.

Despite their usefulness, the sensitivity and reproducibility of liquid-biopsy-based methods using cfDNA still need to be improved, especially in those applications working with a very low amount of starting sample.

Beyond the lack of evidence of clinical utility for these scores, some issues remain in the field, like transethnic applicability [76] and ethical issues, mainly related to genetic data. However, the integrated use of PGS with other omics data in predicting clinical risk and the analysis of their interactions with environmental factors appears to be the road ahead for personalized medicine.

There is the need for large prospective population-based studies with a large amount of data collected with standardized methods, along with biological samples collected and stored in a biobank. In Italy, recently, the Moli-sani cohort study [77] (24,325 subjects aged 35–99 years, recruited between 2005 and 2010 from the general population) started to measure genomic and epigenomic data on biobank samples.

### 2.2. Susceptibility to Environmental Pollution, as Inferred from a miRNA Analysis 

Environmental pollution is a growing public health burden associated with numerous adverse health effects. It is estimated that around 4.2 million deaths occur each year due to air pollution [78]. Biomarkers reflecting specific air pollution exposures have the potential to measure the internal dose resulting from exposure to complex environmental mixtures [79]. MiRNAs, small noncoding RNAs that regulate gene expression, have been studied as biomarkers in various diseases and have also shown potential as biomarkers of environmental exposure [80,81].

In this context, epigenetics bridges the gap between genetic makeup and environmental exposures to pollution, thus explaining the development of diseases [82].

The epigenome is a plastic entity modifiable by the environment, whose changes may be detected in accessible surrogate tissues (e.g., peripheral blood, urine, buccal cells, etc.). These epigenetic changes show long-term stability; accordingly, the epigenetic pattern may be considered a record of environmental exposures experienced throughout life [83]. 

The patterns of miRNA alterations are specifically related to the type of environmental pollution to which the organism has been exposed. In studies where smoking-induced changes were investigated, the general observation was a miRNA downregulation of expression, as initially demonstrated in mouse lungs [84] and thereafter confirmed in human bronchial epithelial cells [85]. MiRNA alterations accumulating during carcinogenesis is a prerequisite for full cancer appearance and progression [80,86,87]. MiRNAs are massively dysregulated during lung carcinogenesis, induced by cigarette smoke [84] but also other environmental airborne lung carcinogens and air pollutants [80]. These early alterations are reflected in the extracellular miRNA released from the lungs in the blood during the different stages of carcinogenesis, including the development of microadenoma, adenoma, and adenocarcinoma [88]. However, the transferability of these results to the human situation is quite controversial. Predictive miRNA signatures of lung cancer have been identified in the blood, but they greatly vary between different studies [88]. Many organs contribute to the miRNA burden in the blood, whereas the contribution of the lungs is negligible as compared to that of the muscles and liver. Indeed, miRNA plays a pathogenic role in cancer only when the target oncogene is mutated, and the extracellular release of miRNA corresponds to a cancer-related event and not to an adaptive response to carcinogen exposure. The shift from adaptation to pathogenic alterations of the miRNA machinery depends on the duration of the exposure to environmental pollution. Indeed, long-term exposure leads to irreversible epigenetic alterations [86]. 

Secondary prevention may be achieved in lung cancer by identifying high-risk individuals using a predictive epigenetic biomarker, such as miRNAs, as analyzed in body fluids. This personalized approach is referred to as “preventive theranostics” and represents an emerging application of personalized medicine to cancer prevention. 

Researchers are currently publishing various miRNA signatures that respond to environmental exposure and have shown usefulness as biomarkers. The relative expression of miRNAs can be studied by various techniques, such as microarrays, used as an initial approach to finding the potential candidate miRNAs, or quantitative real-time PCR (qPCR), used to validate highly dysregulated miRNAs. Moreover, miRNAs can be sequenced and quantified using next-generation sequencing (NGS) platforms [89]. Due to their stability, miRNAs have been extensively studied in the peripheral blood and urine. 

Future research should focus on (a) identifying the molecular mechanism underlying miRNA expression changes in response to environmental exposures; (b) to determine whether the changes in miRNA expression are an early signal of the pathogenic processes developing in the organism; (c) whether or not this miRNA signature may be used for early detection of cancer identifying high-risk subjects (secondary prevention); and (d) whether miRNA alterations are drivers or passengers in the ongoing carcinogenesis process. 

The use of miRNA containing microvesicles is a hot topic in research on the early diagnosis and prognosis of cancer. Given their stability and abundance in blood serum, miRNAs containing micro-vesicles have been proposed as new diagnostic biomarkers for cancer [90]. Extracellular vesicles can transfer information from cell to cell, thus representing a potential mechanism explaining how different environmental exposures interact with the molecular machinery of our organism [91]. Extracellular vesicles play an important role in lung cancer pathogenesis and may have potential as biomarkers [92]. However, the use of extracellular microvesicles as an endpoint in cancer prevention has not yet been fully explored. The association between target-mediated function and miRNA regulation provides a new opportunity for developing novel anticancer preventive strategies, spanning from early diagnosis to the identification of high-risk subjects and prevention of cancer relapses.

### 2.3. Nutritional and Molecular Epidemiology for Precision Prevention and Health Promotion

The Human Genome Project and the increased use of the omics approach in nutrition science have led researchers to focus on personalized nutrition in order to improve the impact of diet on health status and wellbeing, taking into account individual genetic factors and epigenetic signatures [93]. Precision and personalized medicine are useful tools for preventive strategies and could help with predicting morbidity and mortality and detecting chronic disease much earlier in the disease course, to improve the quality of care and quality of life of the patients and reduced healthcare time, efforts, and costs [94]. It is well established that environmentally related diseases are the result of the “exposome”—the totality of exposure experienced by an individual during life and the health impact of those exposures [95]. Epigenetic phenomena can potentially be modified by environmental and lifestyle factors, including diet, and result in environmental reprogramming of the genome for exposed individuals and future generations of offspring [96]. For example, altered expression of miRNA profiles in maternal blood or placental tissue may reflect not only gestational disorders but also prenatal exposure to environmental pollutants and dietary factors [97]. The protective effect of diet and nutrients can be mediated by reversible epigenetic mechanisms, representing an attractive target for health promotion and the prevention of noncommunicable diseases. For instance, hypomethylation of long interspersed nuclear elements (LINE-1), a surrogate marker of global DNA methylation, has been associated with an increased risk of certain types of cancer, although conflicting findings have also been reported [98]. Blood-based methylation biomarkers, which are easier to obtain and adaptable to population screening for the identification of individuals with cancer or those who are at higher risk, are a new research area of great interest. In particular, LINE-1 methylation, together with other differentially methylated regions, has been proposed for the screening and noninvasive early diagnosis of women at risk of cervical cancer [99]. Interestingly, a dietary pattern characterized by low fruit consumption and folate deficiency has been associated with LINE-1 hypomethylation and thus with higher cancer risk [100]. Therefore, public health interventions to change the unhealthiest dietary patterns in favor of the healthiest options may reduce the risk of hypomethylation and, consequently, of cancer. Moreover, specific foods, nutrients, and healthy dietary habits, such as a Mediterranean-style diet and a diet based on the combined intake of nutrients with antioxidant properties, could help to prevent the progression of persistent high-risk human papillomavirus infection to cervical cancer [101,102]. However, other host factors, including genetic polymorphisms, which may explain some of the individual differences in disease occurrence, should be considered since they could be used to target specific and effective preventive strategies [103]. 

Although it is reasonable to expect that, in the future, disease prevention and treatment will be formulated at the individual level according to genomics features, at present, the major challenge for public health genomics is represented by the advance of the scientific evidence necessary to demonstrate if and when the use of genomic information in public health can improve health outcomes in a safe, effective, and cost-effective manner, also taking into account the ethical, legal, and social issues [104]. Further studies will examine epigenetic signatures as biomarkers to identify populations that may particularly benefit from incorporating health behavior changes into plans for precision medicine [105].

### 2.4. The Microbiome of Children: Development and Disease Implications, and Challenges for a Healthy Life 

Human‒microbiota co-evolution also follows an intergenerational transmission pattern [106]. The origin of the gut microbiota in each human is the placenta microbiome before birth, and its composition is then prominently conditioned by the delivery mode [107]. However, after a few months of life, such differences disappear, and other factors become conditioning. During the postnatal period, the environment changes quickly, so parental care is crucial as food transitions occur. A selection favoring host mechanisms is generated, including innate and adaptive immune mechanisms, to control the gut microbiota for the host’s advantage [7]. 

From such a point of view, the gut microbiota is a selective agent shaping the adaptive evolution of the human diet, phenotypic plasticity, gastrointestinal morphology, and immunity. Therefore, as can be expected, microbiota aberrations (dysbiosis), since childhood, have been associated with a range of communicable [108,109,110] and noncommunicable diseases, including obesity and metabolic syndrome [111], diabetes [112,113], inflammatory bowel disease, nonalcoholic fatty liver disease [114], asthma, allergies [115], some types of cancer [116], and even certain neuropsychiatric disorders [117] (Table 1). 

The developmental origins of health and disease provide a theory by which to understand the pathogenic pathways that explain early-stage environmental factors such as human gut microbiome modulation [126]. Exposure, during the highly plastic period of early life, can determine later disease risk. The first three years of life are fundamental for microbiota development.

The intra-individual (α-diversity) and functional complexity increases with age, while the interindividual variations (β-diversity) become less marked. The ratio among the principal observed microorganisms’ families changes with a reduction in the *Enterobacteriaceae* and an increase in the *Bacteriodaceae*. This shift is due to different feeding practices after weaning. The degradation of complex fibers and carbohydrates improves the role of the microbiota in the host metabolism.

The exposure profiles and the gut microbiota change during such a period may then lead to health and disease predisposition in adulthood and can influence aging. Even if a real interconnection and predictive role of the microbiota modulation is not yet clear for most diseases, the clarification of such processes is the key to achieving health improvement for future generations.

From an immunological point of view, various hypothetical models clarify the interconnections of microbiota with the host and the increased prevalence of the disease with an immunological etiology. For example, for type 1 diabetes, five models have been proposed [127], among which there are models with a similar biological plausibility that include the early microbiota development.

From a preventive medicine point of view, knowledge regarding the eubiosis—an equilibrium status that can guarantee the integrity of the gut mucosa—and dysbiosis transitions of the gut microbiota is crucial. The focus could be the identification of validated biomarkers able to describe such a transition. In particular, we need to know about the growth of microorganisms that promote inflammation and the decrease of other groups that can promote host monocytes’ collaboration in human homeostasis. 

The microbiological methodology seems to privilege a global approach to the complexity of the microbiota, but, on the other hand, a simpler approach based on valid biomarker identification is useful for risk stratification in public health.

The chronic inflammation described in older people is characterized by a reduction in microorganisms such as *Bifidobacterium*, *Akkermansia*, and *Christensenellaceae*.

A recurrent question for diseases associated with dysbiosis status is whether the dysbiosis is a determinant, a risk factor, or a consequence of the disease. Nowadays, the evidence seems to suggest very early modification of the microbiota in disease, so its preventive role as, at least, an early detection opportunity before clinical onset, is fundamental for better clinical management and prognosis.

Dysbiosis not only has an effect on the gut but also on distant organs. Microbial metabolites, such as the induction pathway typical of pathogens and to produce pro-inflammatory cytokines, can reach several organs, also inducing neurodegenerative diseases and cancer. Therefore, even when the identification of the point break between eubiosis and dysbiosis has been identified, how should an intervention proceed? 

Different methods have been suggested. The only cost-effective method for the treatment of recurrent vancomycin-resistant *C. difficile* infection is fecal microbiota transplantation. However, such therapeutic intervention—validated from an economical point of view for elderly patients—is not yet common in children because it seems to be associated with a high incidence of adverse effects, including severe adverse effects known in adults (>8%) [128].

Another method consists of the development of a target therapy: for example, using selective antibiotics or phages with the purpose of decreasing the bacteria involved in the pathogenic processes. However, such therapies are uncertain both in terms of the unclear definition of the microbial target and the possible adverse effects [129].

The most common intervention is the intake of a beneficial microorganism probiotic and/or prebiotic, but a wider discussion of the evidence of the beneficial effects is ongoing [8]. The main limitations are a lack of evidence of beneficial effects and a failure to take into account individuals’ peculiarities. The microorganisms most commonly used in the probiotics industry belong to the *Lactobacillus* and *Bifidobacterium* genera. In this field, personal beliefs, intuition, and commercial interests, coupled with a lack of sufficient medical regulation, often combine to make objective interpretation difficult [129]. On the other hand, drug‒microbiome interactions vary between individuals, demonstrating how the gut microbiome has to be included in drug development and personalized medicine [130].

The most acceptable intervention consists of a variation in nutritional behavior, which can shift the microbiota characteristics and lead to an improvement in the balance of the gut microbiota by increasing health-favoring bacteria. A valuable effect is observable in the medium and long term, but only for as long as the subject maintains the healthy diet [8].

Most of the studies on children today are case-control studies and highlight a significant involvement of the microbiota in the risk modulation for various diseases. In general, a decrease in the level of some microorganisms is a recurrent risk factor—for example, *Bifidobacteriaceae*, *Faecalibacterium* spp., *Ruminococcaceae*, *Dialister*, *Roseburia*, and *Akkermansia*. On the other hand, an increase of *Prevotella* and *Enterococcus* leads to greater risk (Table 1). 

The integration of analytic methods, which are not yet standardized for whole-genome sequencing, can provide a more accurate overview of the gut microbiota status. 

The corrections possible today mainly include nutritional and behavioral improvement. Gut microbiota research indicates an enormous avoidable burden of disease, considering years of life lost (or healthy years of life for diseases that originate during childhood), but, today, a consolidation of the evidence is needed as a cost-efficacy evaluation. Moreover, a cohort study with long observation during childhood can elucidate the real contribution of microbiota variation. 

### 2.5. A Precision Medicine Approach in COVID-19 Patients: Which Markers Should Be Used for Prognosis? 

Precision medicine, also known as personalized or stratified medicine, is an emerging paradigm in disease diagnosis, prevention, and treatment [131,132], aiming at targeted treatments tailored to patient characteristics, which include not only biomarkers, but also individual, social, and economic factors [133]. This represents a novel strategy to rapidly identify, in a noninvasive way, an altered biology and to discern the pathways in individuals suffering from a disease, thereby guiding the most appropriate therapy [134,135]. The approach has as its cornerstone the recent advances in omics sciences, molecular biology, and bioinformatics that support the evaluation and treatment of disorders, focusing on four main principles: prediction—anticipating the disease occurrence based on risk factors, lifestyles, and social determinants; prevention—delaying the disease’s evolution before the initial manifestations and once it has settled; personalization—adapting the best therapeutic strategy by analyzing genetic, molecular, and individual factors; and participation—involving biomedical research, academic institutions, health professionals, and the patient [131]. 

In a multisystemic disease process such as an infection, blood (serum and plasma) analysis provides early detection to characterize the damage, with the use of molecular technologies revealing specific biomarkers associated with certain phenotypes/trait groups, leading to therapeutic changes in patients with diverse clinical presentations [134]. Indeed, personalized medicine encompasses the study of disease pathophysiology and the discovery of mechanisms and gene variants [136,137]. Through big data analysis, personalized medicine is effective at recognizing risk factors and biomarkers, and so is valuable for predicting health outcomes and choosing the best treatment and prevention strategies for a particular patient. Novel therapeutic strategies can be addressed by taking into account genetic information (e.g., underlying genes or variants rather than symptoms) in an integrated system [138]. The study of the pathophysiological mechanisms (endotypes) and clinical disease expression (phenotypes) promotes an approach tailored to the characteristics and needs of a patient, with their active participation in the decision-making [136]. Therefore, models of personalized medicine require large genomic databases, a changed methodology involving clinicians, scientists, patients, and the general population, and collective participation [137]. The principles of personalized medicine have been already applied in the so-called fields of vaccinomics and adversomics to help us understand interindividual variations in vaccine-induced immune responses and vaccine-related adverse side effects [139], providing models for profiling the innate, humoral, and cellular immune responses—integrated at a systems biology level to discover vaccine response biomarkers and obtain a directed approach for vaccine development [140]. This knowledge could significantly improve comprehension for individuals who are at risk of such infections, and help determine the type or dose of vaccine needed [135]. 

Since the beginning of 2020, the spread of the severe acute respiratory syndrome coronavirus-2 (SARS-CoV-2) has rapidly led to serious challenges for hospital care [141], and coronavirus disease 2019 (COVID-19) has become an emergent epidemiological threat globally, especially for highly vulnerable population groups [142,143]. The SARS-CoV-2 pandemic has severely affected the world population and global healthcare structures, with first aid and intensive care (ICUs) wards under a degree of pressure never experienced before. Consequently, most of the infrastructural and professional resources have been used to counter this unprecedented emergency. At the same time, traditional screening, outpatient, and surgical activities have slowed down and, in some cases, been completely suspended. The combination of these unfavorable conditions has led to a profound crisis in the traditional model of diagnosis and treatment. In Italy, as in other European nations, the number of cases and deaths dramatically increased starting in March 2020 [144]. On 13 December 2020, more than 1,800,000 confirmed cases have been reported in Italy, with over 60,000 deaths, representing one of the highest mortality rates from the initial diffusion [145,146]. 

Despite the majority of individuals with COVID-19 exhibiting only mild symptoms or even being asymptomatic, there are patients who develop serious complications, underlining the fact that it is crucial to identify who is at higher risk of a worse prognosis and to recognize reliable outcome predictors in a timely manner for improving patient management. As a result, the personalized medicine approach appears to be highly appropriate for the study of COVID-19, considering the wide spectrum of severity and variable phenotypes [147], including asymptomatic, mildly symptomatic, severe symptomatic requiring hospitalization, and respiratory failure due to acute respiratory distress syndrome (ARDS) [148]. Omics-scale studies on SARS-CoV-2 are quickly emerging and have a huge potential to resolve the infection pathophysiology [149], highlighting biologic pathways, modifiable risk factors, and critical information to allow early interventions [150,151]. Hence, comprehension of the underlying mechanisms can be a pivotal step for an individualized therapy [9]. The application of personalized medicine based on the integrated information of the genetic background of COVID-19 patients, individual factors, and clinical data is acquiring great relevance, enabling the detection of predictive biomarkers and paths that are valuable to select specific and effective measures for both prevention and management [132,150,152].

There is growing evidence that COVID-19 occurs more in males [153,154], the elderly, and non-O blood type individuals [155,156]. The inflammatory responses and cytokine storm induced by SARS-CoV-2 are extremely variable [157], and prognosis is conditioned by the host response more than by the infection, since pre-existing comorbidities such as obesity, cardiovascular diseases, arterial hypertension, type 2 diabetes mellitus, and immunosuppression strongly contribute to fatal outcomes [158,159,160,161,162]. 

To date, research focused on the analysis of single-nucleotide polymorphisms (SNPs) of the angiotensin-converting enzyme 2 (ACE2), a receptor of SARS-CoV-2, revealed that morbidity, clinical course, and mortality depend on ACE D allele frequency [163]. Another study investigated the involvement of genetic factors associated with SARS-CoV-2 infection, particularly ACE-related genes [164], showing a negative correlation with the number of cases and number of deaths due to viral infection, since both decreased with an increasing ACE1 II genotype frequency; thus, ACE1 polymorphisms could be useful markers for the prediction of high-risk groups and COVID-19 severity. Moreover, investigating 12,343 SARS-CoV-2 genome sequences from patients in six geographic areas, 1234 mutations were found compared with the SARS-CoV-2 reference sequence, and the frequency of several human leukocyte antigen (HLA) alleles was significantly associated with the fatality rate [165]. In particular, through a hierarchical clustering analysis, 28 countries were grouped into three clusters, with Italy in cluster 2, whose average fatality rate was higher than that of countries in clusters 1 and 3. A genome-wide association study on COVID-19 and a severe disease pattern, defined as respiratory failure, which included seven hospitals in Italy and Spain, found an association signal at the ABO blood type locus, and a specific gene cluster as a genetic susceptibility locus in patients with respiratory failure [166]. Indeed, an analysis showed a higher risk in blood type A than in others (Odds Ratio, OR = 1.45), and a protective effect in blood type O as compared with the other groups (OR = 0.65). So far, the relationship between the ABO blood type and SARS-CoV-2 infection has been supported by other studies [167,168] as being valuable for predicting individual risk, with it being reported that the A blood type may be linked to increased infection susceptibility, in contrast to the O blood type’s protective effect. The ABO blood types have also been associated with different COVID-19 severity patterns, because patients with blood type A or AB were at increased risk for hospitalization, mechanical ventilation, renal replacement therapy, and prolonged ICU admission compared with those with O or B blood types [169,170]. These findings were previously reported for SARS, and likely explained by the presence of IgG anti-A isoagglutinins in subjects with O blood type, preventing the binding of SARS-CoV-2 to its receptor and the virus’s entry into human cells [171]. Consequently, differences in blood type antigen expression could increase or decrease host susceptibility to many infections, modifying the innate immune response and playing a direct role by serving as receptors and/or coreceptors for microorganisms’ antigens [172].

In a study, RNA-Seq and high-resolution mass spectrometry on 128 blood samples from COVID-19 positive patients with diverse severity profiles and negative individuals were used to quantify transcripts, proteins, metabolites, and lipids in a relational database, enabling analysis and correlations between molecules and patient prognoses. A total of 219 molecular features were mapped with high significance for severity, mainly involved into complement activation, dysregulated lipid transport, and neutrophil activation [173].

As COVID-19 is characterized by a variable course, with asymptomatic individuals and others experiencing fever, ARDS, or even death, understanding of the antibody response in subjects with severe compared to mild disease is needed. A high-throughput method was used to analyze epitopes of antiviral antibodies in human sera of 232 COVID-19 patients and 190 controls. Results highlighted epitopes ranging from “private”, recognized by antibodies in only a small number of subjects, to “public”, recognized by antibodies in many individuals, and those with severe COVID-19 exhibited stronger and broader SARS-CoV-2 responses, as well as weaker antibody responses to prior infections [174]. 

In conclusion, considering the rapid evolution of the current epidemiological situation and the need to ensure adequate continuity of care and contain transmission, precision medicine is the key to fighting the COVID-19 pandemic by creating patient-specific treatment programs tailored to individual needs. This novel approach, implying the integration of clinical, lifestyle, genetic, and biomarker information for patient stratification, could enable us to achieve a better understanding of critical disease pathways and more precise and validated phenotypic recognition. Multi-omics systems could provide critical information to better understand SARS-CoV-2 and COVID-19 features, defining the individual genetic predisposition for both infection and course severity. For example, epigenomic and transcriptomic analyses would allow us to characterize changes in tissues involved in COVID-19, and to understand the interaction between SARS-CoV-2 and patient cells as a reaction to viral infection. Data integration would finally enable the identification of biomarkers and therapeutic targets to stratify patients and allow better interventions and decisions. Hence, an understanding of pathogenic pathways and the classification of phenotypes in SARS-CoV-2-infected patients might significantly contribute to the design of effective prevention strategies, mitigation interventions, and personalized pharmacological options in order to guide public health actions and improve the chances of better outcomes.

### 2.6. Health Technology Assessment for Public Health Evaluation of Genetic/Genomic Applications on Genetic Tests 

The existing evaluation frameworks for genetic tests, and genetic/genomic applications in general, mainly rely on two popular evaluation approaches, i.e., the ACCE model (analytical and clinical validity, clinical utility, ethical, legal, and social implications) and the Health Technology Assessment (HTA) process [175]. The U.S. Centers for Disease Control and Prevention created the ACCE model in the early 2000s, specifically for the evaluation of genetic tests [176]. Its name refers to the evaluation dimensions used, i.e., analytic validity (a test’s ability to accurately and reliably measure the genotype of interest), clinical validity (a test’s ability to detect or predict the associated disorder), clinical utility (the risks and benefits associated with a test’s introduction into practice), and ethical, legal, and social implications (ELSI—regarding the safeguards and impediments surrounding the testing process) [177]. Furthermore, the ACCE model includes under the umbrella of clinical utility some contextual issues related to testing delivery, such as economic benefits and organizational aspects. However, the strength of this model lies in considering specific aspects of genetic tests not adequately addressed by standard methods for a technology assessment, particularly analytic validity and clinical validity. For this reason, the ACCE model has been adopted, often with some adaptations, by various entities both in the United States and worldwide [175].

Unlike the ACCE model, the HTA approach was developed to cover all health technologies. However, since its creation in the USA in the late 1960s, some attempts have been made to adjust it for the evaluation of genetic tests. A notable example is a framework proposed to guide the public coverage of new predictive genetic tests in Ontario, Canada, which proposed the following criteria to assess a genetic test: intended purpose, effectiveness, additional effects, aggregate costs, demand, and cost-effectiveness [175,178]. The main innovation of these HTA-based frameworks is the attempt to adopt a service delivery approach, i.e., extending the scope of the assessment beyond the technical and clinical performance of a test to consider the economic and organizational implications of the whole testing service [179]. This approach is necessary to support decision-makers in securing an efficient and equitable allocation of health care resources and services. Nevertheless, HTA evaluations of genetic tests have not yet reached the comprehensiveness typical of general HTA frameworks in the analysis of delivery models.

Based on these findings, a combination of the ACCE model, with its focus on the unique aspects characterizing the genetic tests, and the HTA process, useful to guide provision and coverage decisions, might represent the best approach to the evaluation of genetic tests. Recently, Sapienza University of Rome tried to realize such an integrated approach and proposed a framework distinguished by a dual focus on both the genetic test and its delivery models [180]. The first section of this new framework addresses the genetic test from a technical and clinical perspective, mostly adopting the ACCE evaluation dimensions (analytic validity, clinical validity, and clinical utility), with the addition of personal utility, a dimension that broadly encompasses the nonclinical outcomes the test may have for patients [181]. The second section addresses the analysis of delivery models, defined as the broad context in which genetic tests are offered to individuals and families with or at risk of genetic disorders [182]. The evaluation dimensions proposed in this section, mostly based on the EUnetHTA HTA core model, are organizational aspects, economic evaluation, ELSI, and, in response to the increasing international interest in patient-centered care, patient perspective [183,184]. 

Overall, despite the efforts made, several issues still affect the evaluation of genetic/genomic applications. The first one, mainly related to the delivery model approach, is the generalizability of findings, given the context-dependence of economic and organizational issues. Another challenge is the lack of scientific evidence to use for evaluation, especially translation studies [185]. Finally, even if evidence collection represents the core of any technological assessment, a comprehensive process should also include priority setting, as the resources available for evaluation are inadequate to address the increasing development of new genetic tests, and appraisal, as the evidence collected needs to be summarized in final recommendations to inform decisions.

### 2.7. Fostering the Implementation of Personalized Healthcare by Developing Health Professionals’ and Citizens’ Omics Science Literacy 

Omics sciences can be considered as disruptive innovations that promise a new era of precision health (PH). To fully harness such potential, we envisage some key prerequisites linked to the system’ capacity building: (i) health professionals should be enabled to manage the omics knowledge and applications, and (ii) citizens need to gain sufficient health literacy to understand the potential benefits, limits, and risks of omics technologies concerning their health [186]. 

While several European countries have implemented specific health policies in this field, few countries have integrated public health genomics into the health system offerings [187], e.g., Italy, which since 2010 has included PH as a dedicated pillar in the National Prevention Plans (NPPs) and published in 2013 the first Guidelines on Genomics in Public Health [104]. More recently, the Italian National Innovation Plan of the Healthcare System, based on omics sciences [188], identified educational efforts geared towards professionals, citizens, and decision-makers as a cornerstone for the relevant implementation of omics sciences in healthcare.

Successful personalized healthcare will only be achieved; however, if all stakeholders develop the required awareness of PH, and this can be achieved by improving health professionals’ capacity building and citizens’ literacy.

The integration of the omics innovation into health system policy and practice requires highly engaged and appropriately trained health professionals [186]. A lack of adequate skills or appropriate attitudes among health professionals might be a barrier to the effective implementation of personalized healthcare. Policymakers and public health experts have emphasized the need for a defined set of core competencies and the inclusion of omics concepts into health professionals’ curricula [189]. Several EU countries developed national policies to enhance the preparedness of health professionals and enable the use of omics knowledge for the prevention, diagnosis, and treatment of diseases [190]. 

In Italy, several initiatives were implemented with the aim of achieving better genomics literacy for both health professionals and the general public. Such educational efforts are in line with the goals of the Italian National Innovation Plan of the Healthcare System, based on omics sciences and published in 2017 [188]. Since 2011, in the context of two different projects funded by the Italian Ministry of Health (MoH), two effective distance training courses on genetics and genomics have been released for physicians [191,192]. Other initiatives in this field are currently underway in Italy, directed at a larger audience of healthcare professionals including biologists, pharmacists, and other professional categories (https://www.eduiss.it/) [193].

With the rapid advances in omics technologies, the demand for well-trained healthcare professionals will grow exponentially, and omics education will need to evolve to keep up with the changing scientific landscape. Therefore, for effective and successful implementation of PH, improving health professionals’ literacy will need to be a priority, along with suitable common principles, appropriate policies, and regulatory frameworks. 

Among the great challenges that should be addressed to allow for the correct implementation and integration of omics sciences into healthcare practice, citizens’ engagement and literacy will play a key role [186]. Citizens are expected to adopt new behaviors in this novel healthcare era, including being involved in shaping and developing personalized healthcare, contributing to research, and being engaged in citizen health projects [194].

A striking example of the potential impact of omics technologies on citizens’ health and healthcare systems is direct-to-consumer genetic tests (DTC-GTs). The increasing demand for DTC-GTs among laypeople, together with their peculiar provisional model—i.e., not requiring counseling by health professionals—need not only a careful analysis of the potential benefits and risks by policy-makers [195], but also informed citizens who can be aware of them and, consequently, make appropriate decisions about their health [192]. For these reasons, citizens’ literacy in omics sciences, which is poor according to the literature [196], should be increased using appropriate and effective initiatives and strategies. This is what has emerged from a recently published systematic review of the literature [196]. The study summarizes the current knowledge of citizens’ literacy, attitudes, and educational needs in omics sciences, underlining the need for strengthening public engagement on this topic. In the Italian context, this has been addressed by authorities through several policy documents [104,188] and different projects funded by the MoH. In fact, as part of one of these projects, a survey conducted in collaboration with a citizens’ organization is currently underway, aimed at assessing the real knowledge of Italian citizens about the main issues related to genomics in health.

This further underlines the importance of citizen engagement and literacy in this field, which would allow them to adopt a more active role in the protection of their own health and in shaping more effective strategies for the implementation of personalized healthcare [186].

Several strategic actions will need to be taken to allow for the easy integration of omics knowledge and technologies into healthcare [186].

We could envisage among the main actions:

- developing awareness among stakeholders;
- improving citizens’ health literacy to fully empower them;
- fostering health professionals’ skills acquisition through extensive educational initiatives in omics sciences;
- shaping sustainable healthcare through the use of evidence-based tools such as a Health Technology Assessment for the omics technologies’ evaluation to introduce in healthcare systems.

### 2.8. The Point of View of the Territorial Department of Prevention and the Community Health District 

The Departments of Prevention (DP) are parts of the Local Units of the Italian National Health Service, which oversee public health activities. Their main duties are health promotion, disease prevention, livestock and pet health, and food safety. They promote public well-being by promoting healthy behaviors, preventing infectious and chronic diseases, and improving work safety. They are involved in the strategic planning and evaluation of preventive programs as essential ways to achieve health-related goals. The Direction of the DP guarantees comprehensive governance and integration among the different activity areas and is expected to coordinate and organize public health initiatives [197].

The National Prevention Plan (NPP) it subdivided into regional and local prevention plans, which play a key role in the governance of public health programs. There are some characteristics that public health programs have in common: they

1. are addressed to healthy people in large numbers;
2. represent “proactive” medicine;
3. provide cost-effective and evidence-based technologies;
4. deliver free or co-payment health care services;
5. consider individual as well as community health gain; and
6. are provided in all regions of Italy, as they are mandatory.

These programs are implemented in the following fields:

1. cancer screenings
2. vaccination campaigns
3. risk communication, counseling, health literacy, and empowerment of the target population
4. epidemiological evaluation of the health efficacy in the target population
5. health surveillance activity
6. infectious disease (nowadays, especially COVID-19)

In general, a public health intervention:

- must be efficacy- and value-based;
- needs dedicated resources (personnel, places, technology, software, etc.);
- needs a structured plan from prevention to treatment;
- must avoid inequalities; and
- requires people’s advocacy and involvement.

The same fundamental principles can also be applied to the field of “predictive medicine”, which, in the public health sector, can be implemented by the DP [198]. Predictive medicine should become an integral part of the duties of the DP, as they have the appropriate methodology, experience, attitudes, and trained personnel. The findings of the basic research should be translated into public health programs. Practical written evidence-based guidelines are essential, as well as an evaluation frame to assess the impact on the population. 

The integration of genomics into public health should achieve these results:

- generating more specific and cost-effective public prevention programs;
- enhancing the impact of prevention and risk-reduction campaigns;
- favoring the exchange of information between various branches of the public health sector; and
- maintaining the importance of a central public health author even if the trend is toward personalized medicine.

The NPP 2010–2012 underlined the importance of predictive medicine and its huge potential to identify a population of healthy individuals who are at risk of developing specific diseases [199]. This could provide effective interventions that are specific and personalized. This is in line with the new NPP 2020‒2025, which aims to consolidate the focus on the single individual [200]. This can be achieved by targeted interventions to improve health literacy, enhancing the empowerment of individuals to trust and communicate with the public health sector (engagement). The new NPP also claims that, for nontransmissible chronic diseases, it is necessary to combine and integrate a community-based strategy (such as by promoting healthy lifestyles) and personalized strategies (by identifying people at risk or in the early stages of disease).

Therefore, it is necessary to enhance the understanding of predictive medicine and how it can be developed by the DP. Discussion and implementation have just started. To date, predictive medicine has only found a few applications in Italy. Genomic predictive tests are used in public health settings only to investigate monogenic disorders. These include screening for high-penetrance mutations such as breast, ovarian, and colorectal cancer. Genetic screening for complex diseases has been applied to a very limited number of conditions. For example, the NPP 2014‒2018, based on the public health genomics approach, aimed to develop organized paths of breast cancer prevention in women with BRCA1/2 mutations [201]. This screening is complementary to the ongoing cancer screening program.

On the other hand, large-scale prevention programs that target large population groups (such as vaccination campaigns, cancer screenings, and cardiovascular disease progression monitoring) are already part of the system and have been used for several years. They can constitute essential background to the introduction of predictive medicine in routine activities of DP [202].

There are many reasons to think that the role of the DP could be central and appropriate:

- consolidated and experienced activity of screening;
- an existing network with clinical disciplines;
- experience of risk communication and counseling;
- experience of follow-up management;
- an appropriate attitude toward the analysis and evaluation of prevention activities;
- appropriate software in use.

In conclusion, predictive medicine in the context of cancer screening is already feasible, but it would require an adaptive process to enhance the knowledge and practical organization. DP healthcare workers will have to increase their specific competences. However, these are often already used in daily activities like screening and health promotion, but should be updated with genomics knowledge. On the other hand, vaccine activities may also profit from genomic screening by taking advantage of the stratification of populations and the identification of nonresponders based on their genetic profile. Additionally, the recent COVID-19 epidemic might open up new uses for predictive medicine to identify individuals at high risk of developing a severe condition.

The “*Plan for innovations in the health system based on omics sciences*” (published in 2018) defined ways to improve the health system through the application of omics science [203]. This innovation regards prevention, diagnosis, and care based on efficacy and value for the improvement of individual health. The DP has the tendency to lead to changes in the practical organization of all the units of the Italian NHS involved, helping to define policies for the best use of genomics and omics sciences [204].

## 3. Conclusions

The omics sciences offer a wide range of tools to improve public health: from a single polymorphism detection to the PGS approach, and including whole-genome and exosome sequencing. The interaction between a gene and the environment can be defined by the epigenetic end-point and also by the evaluation of the microbiome shift from eubiosis to dysbiosis, highlighting several prevention opportunities, especially for the early detection of diseases or at-risk conditions. However, the introduction and accessibility of such tools are not yet guaranteed in Italy and they are provided by private bodies, individuals, and sporadic ventures. 

In the future, disease prevention and treatment should be formulated at the individual level according to genomic features. However, a current major challenge is a lack or scarcity of scientific evidence, as well as a lack of ethical, legal, and social regulations. 

The implementation of omics advancements into clinical practice depends on countries’ ability to adopt relevant strategies and innovative approaches. The production, integration, and use of genetic/genomic information in healthcare require significant changes in the way such care is organized and provided to individuals. This is also evident in relation to the COVID-19 pandemic. Therefore, new health policies and specific programs on the omics sciences will be needed to respond to the needs of citizens and all health stakeholders.

## Figures and Tables

**Table 1:** Examples of microbiota modulation involved in disease pathways in children.

Disease	Age	No. of Participants	Study Design	Results	Biological Plausibility	Author, Year
*Clostridium* *difficile* infection (CDI)	11 months–23 years old	372 patients (31 were excluded because they had fewer than 60 days of follow-up, six because of refractory CDI)	Cohort study	Fecal Microbiota Transplantation (FMT) had a successful outcome in CDI pediatric patients: 81% had a successful outcome following a single FMT and 86.6% had a successful outcome following a first or repeated FMT; 4.7% had a severe adverse event during the three-month follow-up period, including 10 hospitalizations	There were four independent predictors of FMT success: - fresh donor stool (during a freeze‒thaw cycle there may be alterations in the viability of critical taxa for the pediatric population) - delivered by colonoscopy (colonoscopy permits us to identify additional comorbidities that often confound the diagnosis of CDI) - lack of a feeding tube, which usually is a risk factor - fewer episodes of CDI recurrence	[108]
*H. pylori-*induced gastritis	4–14 years old	154 (52 *H. pylori*-induced gastritis (HPG), 42 *H. pylori*-negative gastritis (HNG), 62 healthy control (HCG))	Case-control study	Changes in F:B ratio, an increase of *Bacteroidaceae* and *Enterobacteriaceae*, and a decrease of *Lachnospiraceae* and *Bifidobacteriaceae* can be caused by gastritis itself and exacerbated by *H. pylori* infection. These changes may be related to drug resistance and the development of chronic gastrointestinal diseases.	Most of the significant taxa belonged to the Gram-negative bacteria producing LPS. The LPS from the intestinal microbiome induces a chronic subclinical inflammatory process. The upregulation of pro-inflammatory cytokines and downregulation of anti-inflammatory cytokines may be a way to influence gastritis. *Lactobacillus* can change the pH of the intestinal environment to inhibit the growth of pathogenic bacteria and stimulate an immune response.	[109]
Tuberculosis	<14 years old	36 (18 diagnosed or probably infected + 18 healthy controls)	Case-control study	Pulmonary TB patients presented an upregulation of *Prevotella*, *Enterococcus*, and a reduction of *Ruminococcaceae*, *Bifidobacteriaceae*, and *F. prausnitzii.*	*Prevotella* is a pro-inflammatory bacterium that may activate inflammatory reactions that aggravate TB. *Enterococcus* is a pathogen associated with intestinal permeability. The translocation of this bacteria into systemic circulation induces an immune-inflammatory reaction. *F. prausnitzii* is an SFCA producer and SCFAs regulate intestinal permeability. Alterations in *Bifidobacteriaceae* may be associated with a reduction in the immune response against the invasion of foreign microbes.	[110]
Recurrent respiratory tract Infections (RRTI)	Under five years old	49 (26 patients and 23 healthy controls)	Case-control study	ROC analysis: *Enterococcus* achieving AUC values of 0.860	Changes in the gut microbiome’s constituents, with an increased incidence of opportunistic pathogens like *Enterococcus*, are linked to altered immune responses and homeostasis in the airways.	[118]
Intestinal ischemic injuries	<14 years old	14 patients + 9 healthy controls	Case-control study	Enterobacteriaceae’s and *Veillonella dispar*’s increase and a reduction in *Akkermansia muciniphila* might be investigated as a target of intestinal injuries in neonates.	*Enterobacteriaceae* may be related to a pro-inflammatory response by the immature immune system, resulting in homeostasis disruption. *A. muciniphila* stimulates in mice the proliferation of Treg cells and is observed in patients with inflammatory bowel disease, suggesting it may have anti-inflammatory properties. Instead, *V. dispar* has pro-inflammatory effects.	[119]
Nonalcoholic Fatty Liver Disease (NAFLD)	8–17 years old	124 (87 biopsy-proven NAFLD, 37 obese controls) N.B. NAFLD patients were more likely to be male	Case-control study	*Prevotella* was more abundant in children with NASH or obesity.	*P. copri* is the dominant *Prevotella* species. Data analysis showed that *P. copri* abundance was the best predictor of fibrosis severity. *P.* *copri* increases intestinal inflammation to its advantage. Such pro-inflammatory effects may exacerbate liver damage.	[114]
Obesity	111 children aged 6–11; 61 adolescents aged 12–18	172 (76 normal-weight and 96 obese individuals, of whom 46.88% were affected by metabolic syndrome)	Case-control study	Obese children had a higher relative abundance of *Firmicutes* and *Actinobacteria* and decreased *Bacteroidetes*.	*Coriobacteriaceae* family positively correlates with intrahepatic levels of triglycerides and non-HDL plasma concentrations, suggesting an effect on the gut barrier. *Prevotella* is associated with chronic inflammation. *Firmicutes* phylum: *Lactobacillus* is associated with weight gain.	[111]
Type 1 diabetes mellitus (T1D)	Under 18 years old	15 T1DM + 15 nonautoimmune diabetes + 13 healthy controls	Case-control study	Gut microbiota in T1D differs at the taxonomic and functional levels in comparison with healthy subjects and nonautoimmune diabetes patients. T1D was characterized by an increase in *Bacteroidete* and pro-inflammatory bacteria, and a decrease in *Faecalibacterium* and *Roseburia*.	The T1D gut microbiota profile was associated with a loss of epithelial integrity, low-grade inflammation, and autoimmune response, allowing luminal antigens to escape from the gut and promote islet-directed autoimmune responses. The gut microbiota from patients with T1D was significantly enriched with genes for antigen presentation, chemokine production, LPS biosynthesis, and bacterial invasion.	[112]
	2 months‒6 years old	44 children with a first-degree family history of T1D; 22 were exposed to oral insulin and 22 to a placebo.	Cohort study	There are differences in microbiome composition between children with susceptible and nonsusceptible *INS* genotypes, and after oral insulin treatment in children with the susceptible *INS* AA genotype.	There is an increased abundance of *Bacteroides dorei* in children with the susceptible *INS AA* genotype. There is an increased alpha diversity in children treated with oral insulin, who showed an antibody response compared with those without a response; this observation is consistent with a microbiome-mediated treatment effect.	[120]
	5–10 years old	40 T1D patients and 56 healthy children	Case-control study	Modulation of the T1D risk includes higher *Firmicutes* levels (OR 7.30; IC 2.26–23.54) and a greater amount of *Bifidobacterium* in the gut (OR 0.13; IC 0.05–0.34)	The origin of the disease process was suspected to be gut microbiota dysbiosis, associated with altered gut permeability and a major vulnerability of the immune system.	[113]
Juvenile idiopathic arthritis (JIA)	1–16 years old	39 JIA diagnosed patients + 42 healthy controls	Case-control study	The relative abundance of four genera, *Anaerostipes*, *Dialister*, *Lachnospira*, and *Roseburia*, decreased significantly in the JIA group. 12 genera were identified as potential biomarkers (AUC = 0.7975): *Bifidobacterium*, *Lachnospira*, *Dialister*, *Roseburia*, *Oscillospira*, *Akkermansia*, *Clostridium*, *Faecalibacterium*, *Bilophila*, *Coprococcus*, *Haemophilus*, *Anaerostipes.*	*Anaerostipes*, *Dialister*, *Lachnospira*, and *Roseburia* in JIA patients decreased, three of which are butyrate-producing microbes; *Dialister* is a propionate-producing microbe. SCFAs have considerable immunomodulatory effects (inducing the differentiation of regulatory T cells, enhancing IL-10 production, and suppressing Th17 cells; butyrate administration suppressed the expression of inflammatory cytokines).	[121]
Asthma	2–12 months old	618 children for bacterial 16S rRNA 189 children for fungal ITS region	Case-control study	There is an inverse association of asthma with the measured level of fecal butyrate (OR = 0.28 (0.09–0.91), *P* = 0.034), bacterial taxa butyrate producers (*Roseburia* and *Coprococcus*, OR = 0.38 (0.17–0.84), *P* = 0.017) and the relative abundance of the gene encoding butyryl–coenzyme A (CoA): acetate–CoA-transferase, (OR = 0.43 (0.19–0.97), *P* = 0.042). Children who had grown up on farms had a lower risk of asthma compared to others (OR = 0.56).	Butyrate is the main source of energy for colonic epithelial cells; it contributes to the maintenance of the epithelial gut barrier and has immunomodulatory and anti-inflammatory properties.	[115]
Obstructive sleep apnea syndrome (OSAS)	2–12 years old	16 (divided between patients and healthy controls)	Case-control study	*Faecalibacterium* decrease in children with severe grades of OSAS.	*Faecalibacterium* is involved in the production of butyrate, which improves the gut barrier function, upregulating mucin-associated genes in gut goblet cells and the expression of the tight junction proteins.	[122]
Autism spectrum disease (ASD)	2–6 years old	16 ASD children + 7 controls	Case-control study	Gut microbiota decreased biodiversity: four of the 82 GO terms have a role in the catabolic process of the 3,3phenylpropionate mapped to the *E. coli* group.	3,3phenylpropionate is the conjugate base of 3-phenylpropionic acid deriving from PPA. PPA is an SCFA produced during the bacterial fermentation of carbohydrates. The elevated concentration of propionate metabolites could be due to their reduced degradation because of the *E. coli* drop.	[116]
	3–7 years old	78 ASD children + 58 controls	Case-control study	Nine genera and the abundance of seven metallic elements are altered in ASD children. These were used in a diagnostic model in Chinese children with high accuracy (84%).	The diagnostic model is composted by bacterial genera (*Bacteroides*, *Parabacteroides*, *Sutterella*, *Lachnospira*, *Bacillus*, *Bilophila*, *Lactococcus*, *Lachnobacterium*, and *Oscillospira*) and metallic elements (Pb, As, Cu, Zn, Mg, Ca, and Hg). *Parabacteroides* and *Oscillospira* changes could be induced by heavy metal exposure.	[123]
	2–8 years old	43 ASD children (19 with GI symptoms and 24 without) + 31 controls	Case-control study	34 MEs (gut microbiota-associated epitopes) are a potential biomarker of ASD. Those alterations may contribute to abnormalities in gut immunity and/or homeostasis in ASD children.	29 of 34 MEs decreased and were associated with abnormal gut IgA levels and altered gut microbiota composition;11 of 29 were pathogenic microorganisms’ peptides with T or B cell response. ME with homology to a Listeriolysin O peptide from the pathogenic bacterium *Listeria monocytogenes* is increased.	[124]
Acute lymphoblastic leukemia (ALL)	2–25 years old	51 (23 matched patients and a healthy sibling and five unmatched patients)	Case-control study	It was possible to distinguish between the patient and control groups based on their microbiota profiles. *Lachnospiraceae* (which comprises the *Clostridium XIVa*) and *Roseburia* are butyrate-producing bacteria and were greatly reduced in acute leukemia patients compared to a healthy sibling; instead, *Bacteroides* increased.	Bacteria producing butyrate play a major role in the composition of the mucus layer, as butyrate is an important energy source for intestinal epithelial cells and plays a role in the maintenance of colonic homeostasis. Butyrate-producing bacteria may increase the risk of developing chemotherapy-induced mucositis and other GI complications. Antibiotic-induced shifts can increase the susceptibility to *C. difficile* infection.	[125]
Rhabdomyosarcoma	3–7 years old	3 oncologic patients + 2 healthy controls	Case-control study	After radiation exposure, there was an increase in α-diversity related to nonresponsive radiotherapy treatment, and a decrease in *Firmicutes*, associated with a *Proteobacteri*a increase. This information could be used for the definition of the therapy.	The decrease of *Firmicutes* could explain the variation in α-diversity and the ability to survive of the *Proteobacteria* phylum and might be related to DNA mutations.	[117]
